# Institutional Outdoor Work and Administration

**Published:** 1911-09-09

**Authors:** 


### INSTITUTIONAL OUTDOOR WORK AND ADMINISTRATION.

### III.—HOME SUPPLY OF PORK AND BACON.

Although it may confidently be asserted at the outset that this is a vastly simpler and more profitable matter than might be supposed, none the less it will be well first of all to count the cost and the possible margin of profit. The cost will vary, however, according to the circumstances of particular cases, and so can only be stated here generally. The amount of waste food available from the institution itself is one great factor, for pig-keeping, so experts say, does not pay where all the food is to buy. This dictum refers, of course, to the usual case, where pigs are sold in the open market, not to our present and rather unusual one, where all middleman profits are eliminated by the carcase being taken straight from the sty to the kitchen. Such profits mount up considerably, for, to begin with, there are the cost of carriage to market and the auctioneer's fees; or, if pigs are sold through the pig-man by private treaty, there is that individual's little secret commission on each, which surcharge cannot in practice by any means be checked. Then, again, pork which a butcher buys at sixpence per pound he will sell again at ninepence.

If it be only contemplated to breed pigs for selling in this manner, or to buy young pigs and fatten them, under the most favourable conditions the full return cannot be looked for. It is much better to get the breeder's profit as well, to (literally) go the whole hog, and breed, rear, and kill on the premises. Pigs are prolific animals and require hardly any obstetric attention. They are managed with exceedingly little trouble, a mere tithe, indeed, of that necessary with poultry.

As a first step, the organiser of this little enterprise should get Professor Long's *Book of the Pig*, and Mr. Garrett's *Practical Pig-keeping*, works* which can both be read through in two or three evenings. The directions there given are simple enough, but can in this respect be further improved upon in practice. There is really no need, in a small self-contained piggery which has no communication with the outside world and can thus scarcely be exposed to infection, for elaborate housing like glazed tiles and iron troughs. The one essential is a concrete floor for yards and sties both. Otherwise the driest and firmest site would soon be "poached" into mire. One part of "hearth cement," mixed with two parts of sand, and laid two inches thick on a foundation of stone and clinker, will provide the floor, which, in the case of the breeding sties, should be covered temporarily with boards at the time of parturition. Then most useful things are empty petroleum barrels, to be obtained at three shillings each from the grocer. Fill with shavings and ignite these to burn out the dregs of the oil, and the barrel is ready for use. Saw off the upper fifth or so of some of these, and sink the larger portions in the ground so as to act as a cesspool to drain the yard attached to each sty. The liquid contents are baled out regularly with a bucket and poured on to the manure heap, which forms a not inconsiderable asset of pig-keeping, or taken straight to the garden. The smaller portions form good feeding tubs for young pigs. Reserve three barrels for keeping food in, merely knocking out the heads and fitting with lids. One is fixed near a steam-pipe, which is led into it and arranged in a perforated coil round the bottom. The refuse tubs from the kitchen are emptied straight into this (all bones being previously crushed in a green bone-cutter), and then by adding water and turning on the steam the food is at once sterilised and cooked. Boil everything, and all objections as to possible conveyance of tuberculosis (which, in any case, would seem from the results reached by the recent Royal Commission to be a quite unlikely occurrence) are obviated. The above arrangement, which can be fixed in half a day by any engineer, is the one in use at a certain large asylum which boasts a particularly large piggery. The two other barrels are kept near the sties and filled from the boiler. Fattening pigs need as well a little meal, and all pigs kept shut up should have grass sods and some coal or cinders.

So much for food and accommodation. As regards the breed kept, a common kind at institutions seems to be the pedigree Middle White. It is hardy, readily eats grass, fattens very—almost too—easily, and is docile. As to the number, a community of about sixty persons will consume a side and a half of bacon in a fortnight, or, say, forty bacon pigs a year. Add fifteen porkers of about 60 lb. deadweight each, and we thus get a total of just over a pig a week, on an average, to be raised. Four brood sows are needed for this, so four breeding sties (duly fitted with a rail round the sides) must be constructed. Larger separate pens for store pigs and fattening pigs, to hold, say, ten of each, are needed, and away from all these, and more strongly built, a boar pen. It is well worth while to keep one's own boar, if only that the animals available in the neighbourhood are nearly always coarsely bred. Then the time and trouble of travelling sows to these are considerable. Besides, in a little while stud fees may be obtained from the local pig-keepers, who will soon see the advantage of the produce of well-bred stock. The boar should be of a larger variety. At the Edinburgh Farm Colony for Consumptives may be seen a fine Large White boar, and his stock certainly combine admirably the size of the sire with the fattening qualities of the dam. In conclusion, pigs are no trouble and by their comicality appeal to all animal lovers. Under suitable conditions they are most profitable; in the above particularly advantageous case, indeed, after paying a pig-man a sovereign a week (seeing that a great deal of his time will be left at liberty for other work) it should be possible to save the commissariat account £100 a year.

*(To be continued.)*

* Although the first of these recommends pig-ringing forceps, curiously enough neither of them states where to get these useful appliances. The name of a good maker happens to be a familiar one, that of Messrs. Arnold, the well known instrument makers, near St. Bartholomew's Hospital.

